# Harnessing Bacterial Extracellular Vesicle Immune Effects for Cancer Therapy

**DOI:** 10.20411/pai.v9i1.657

**Published:** 2024-04-23

**Authors:** Irem Karaman, Asmita Pathak, Defne Bayik, Dionysios C. Watson

**Affiliations:** 1 Bahcesehir University School of Medicine, Istanbul, Turkey; 2 Sylvester Comprehensive Cancer Center, Miller School of Medicine, University of Miami, Florida

**Keywords:** bacterial extracellular vesicles, cancer immunotherapy, bioengineering, biomarker, vaccine, drug delivery, targeting, drug development, clinical translation

## Abstract

There are a growing number of studies linking the composition of the human microbiome to disease states and treatment responses, especially in the context of cancer. This has raised significant interest in developing microbes and microbial products as cancer immunotherapeutics that mimic or recapitulate the beneficial effects of host-microbe interactions. Bacterial extracellular vesicles (bEVs) are nano-sized, membrane-bound particles secreted by essentially all bacteria species and contain a diverse bioactive cargo of the producing cell. They have a fundamental role in facilitating interactions among cells of the same species, different microbial species, and even with multicellular host organisms in the context of colonization (microbiome) and infection. The interaction of bEVs with the immune system has been studied extensively in the context of infection and suggests that bEV effects depend largely on the producing species. They thus provide functional diversity, while also being nonreplicative, having inherent cell-targeting qualities, and potentially overcoming natural barriers. These characteristics make them highly appealing for development as cancer immunotherapeutics. Both natively secreted and engineered bEVs are now being investigated for their application as immunotherapeutics, vaccines, drug delivery vehicles, and combinations of the above, with promising early results. This suggests that both the intrinsic immunomodulatory properties of bEVs and their ability to be modified could be harnessed for the development of next-generation microbe-inspired therapies. Nonetheless, there remain major outstanding questions regarding how the observed preclinical effectiveness will translate from murine models to primates, and humans in particular. Moreover, research into the pharmacology, toxicology, and mass manufacturing of this potential novel therapeutic platform is still at early stages. In this review, we highlight the breadth of bEV interactions with host cells, focusing on immunologic effects as the main mechanism of action of bEVs currently in preclinical development. We review the literature on ongoing efforts to develop natively secreted and engineered bEVs from a variety of bacterial species for cancer therapy and finally discuss efforts to overcome outstanding challenges that remain for clinical translation.

## MICROBIOME REVELATIONS SPUR INTEREST IN BUGS-AS-DRUGS FOR CANCER

The human microbiome consists of the bacteria, fungi, viruses, archaea, and protozoans that symbiotically co-exist in and on the body [1, 2]. Commensal microbes have been detected in increasing anatomic locations outside the prototypical microbiome of the gut, including skin [3], lungs [4], genito-urinary tract [5], and blood [6]. As an integral part of organism homeostasis, the microbiome impacts endocrine function [7, 8], cardiovascular function [9], immune regulation [10], nutrient digestion [11], and even drug metabolism [12]. As a result, microbiome dysregulation, or dysbiosis, is being associated with a wide variety of diseases and clinical outcomes, including cancer [13, 14]. Following early preclinical results suggesting that an intact microbiome is critical to immunotherapy responsiveness [15], clinical studies demonstrated that the specific composition of the microbiome is associated with response and resistance to immune checkpoint inhibitor therapy across several cancer types [16–18]. Moreover, healthy donor fecal microbiome transplantation combined with PD-1 inhibition demonstrated efficacy in melanoma patients refractory to PD-1 inhibitor monotherapy [19, 20].

The microbiome's effect on cancer immunotherapy highlights the promise of developing microbiome-based therapeutics and their value as potential biomarkers. However, the limited understanding of mechanisms that regulate host-microbiome interactions remains a challenge, despite increasing numbers of studies showing a correlation between the composition of the gut microbiome and clinical outcomes. *Akkermansia mucinophila* [21]*, Bifidobacterium longum* [22]*, Ruminococcaceae* [16], and a high Firmicute-to-Bacteroidia ratio [16, 23] have all been linked to checkpoint inhibitor response in various cancers. One proposed mechanism for these observations is the effect of microbe-derived secreted metabolites on immunity upon absorption through the gut mucosa [24]. For instance, acetate produced by *Bifidobacterium bifidum* [25] and the short-chain fatty acid butyrate [26] were shown to enhance anti-tumor immunity by increasing tumor-infiltrating IFNγ secreting CD8+ T cells. In addition, a series of studies has identified microbes within solid tumors, which are associated with immunotherapy response [27–29]. While there has been some debate [30] regarding the validity of studies that rely on sequencing data to characterize tumor microbiomes [31], several studies have employed orthogonal approaches (including histological analyses and culturing of live tumor-derived bacteria) to support the presence of viable microbes within tumors [27, 29]. As understanding of the rules governing tumor microbe colonization and the molecular basis of their effects increases, opportunities may arise for relevant diagnostics and therapeutics in this area as well.

Besides metabolites and viable bacterial cells, bacterial extracellular vesicles (bEVs) comprise an additional bioactive component of the microbiome. These nano-sized (~20 to 200 nm) vesicular structures contain biologically active macromolecules (lipids, glycans, proteins, nucleic acids) and are produced by essentially all bacteria [32–34]. The role of microbiome-derived bEVs in modulating immunity in cancer is only just beginning to be explored, with most studies focusing on effects bEVs have on cells in the lamina propria of the gastrointestinal tract [35–37]. Proof-of-principle studies have demonstrated that bEVs administered systemically can modulate the immune microenvironment of tumors and affect treatment response [38–40]. These studies have raised significant interest in bEVs as potential therapeutics and biomarkers.

In this review, we first present an overview of established literature describing the immune effects of bEVs in various disease contexts and at barrier sites, as data suggests that this comprises the primary mechanism of action of therapeutic bEVs. We then discuss how bEVs are being developed as potential cancer therapeutics in preclinical studies. Future studies on the mechanisms governing bEV-host interactions will be instrumental in the development of relevant biomarkers and therapeutics.

### Bacterial EVs Modulate Innate and Adaptive Immunity

Ongoing research suggests that the generation of bEVs is a tightly controlled mechanism characterized by the inclusion of specific biologically active components [41]. These bEVs serve as vectors for bacteria-bacteria and bacteria-host communication by interacting with recipient cell receptors, delivering biological macromolecules, and incorporating them into the host cell cytoplasm [33]. Through these interactions, bEVs mediate intracellular signaling, horizontal gene transfer, virulence factor delivery [11, 42–45], and immune modulation [46]. Three primary mechanisms for the effects of bEVs have been suggested: endocytosis, membrane fusion, and receptor-mediated signaling. The kinetics of bEV-host cell entrance is governed by species-specific composition, such as the presence of specific structures of lipopolysaccharides (LPS) in gram-negative bEVs [11, 47–49]. The bEVs from some species, such as those produced by *Aggregatibacter actinomycetemcomitans*, have also been shown to be internalized by cells and sorted to a peri-nuclear localization through clathrin-mediated endocytosis [50]. On the other hand, bEVs with O-antigens, such as those produced by *Escherichia coli*, can enter via lipid raft-dependent endocytosis subsequent to toll-like receptor (TLR) recognition [49].

Until recently, research on bEVs has mostly concentrated on outer membrane vesicles generated by gram-negative bacteria by membrane blebbing. Gram-positive bacteria, which lack an outer membrane, were initially thought likely to be incapable of EV release due to their strong peptidoglycan cell walls [51]. A renewed focus on the formation of these vesicles has been sparked by the discovery of physiologically active EVs in gram-positive bacteria in more recent investigations [51–53]. Structural variations between gram-negative and gram-positive bacteria induce distinct EV biogenesis pathways. Three main theories have been put forward to explain how gram-negative bacteria release extracellular vesicles: genetic mutations that reduce the strength of the outer membrane-peptidoglycan bond, stress responses that cause the accumulation of envelope components, and changes in the stability of the outer membrane caused by interactions with LPS [46]. Gram-positive bacteria form vesicles by budding through the cytoplasmic membrane and entering the cell wall. The synthesis of EVs in gram-positive bacteria is assumed to be influenced by factors such as penicillin-binding proteins (PBPs) and autolysins, as well as genetic factors. As illustrated by Wang et al (2018) [53], specific mutations in penicillin-binding proteins and autolysin have been observed to impact EV production, quantity, and size in *Staphylococcus aureus*, where EV release is correlated with the degree of peptidoglycan cross-linking. This provides more evidence that gram-positive bacterial species may exhibit substantial variation in the EV production process.

In-depth proteomic and biochemical analyses have revealed that bEVs, like their parent bacteria, transport a variety of cargo, including immunostimulatory ligands such as membrane-bound and periplasmic pathogen-associated molecular patterns (PAMPs) [54], enzymes, toxins [55], polysaccharides [56], nucleic acids (DNA and RNA) [57], LPS, lipoteichoic acid (LTA) [58], and peptidoglycan [59]. Both commensal and pathogenic bEVs have been shown to modulate the host immune system via PAMPs that are recognized by host pattern recognition receptors (PRRs) such as TLRs and nucleotide-binding oligomerization domain-containing protein 1 (NOD1) [11]. The different biogenesis pathways during vesiculogenesis, the distinctive membrane envelope structure of their parental bacterium, the genetic make-up of the producing strain, and growth conditions all contribute to the heterogeneity of bEVs in terms of their structure, size, density, and molecular cargo composition. These properties in return have implications on vesicle binding, kinetics in tissues, immunologic effects, and other biological functions [60].

**Figure 1.**
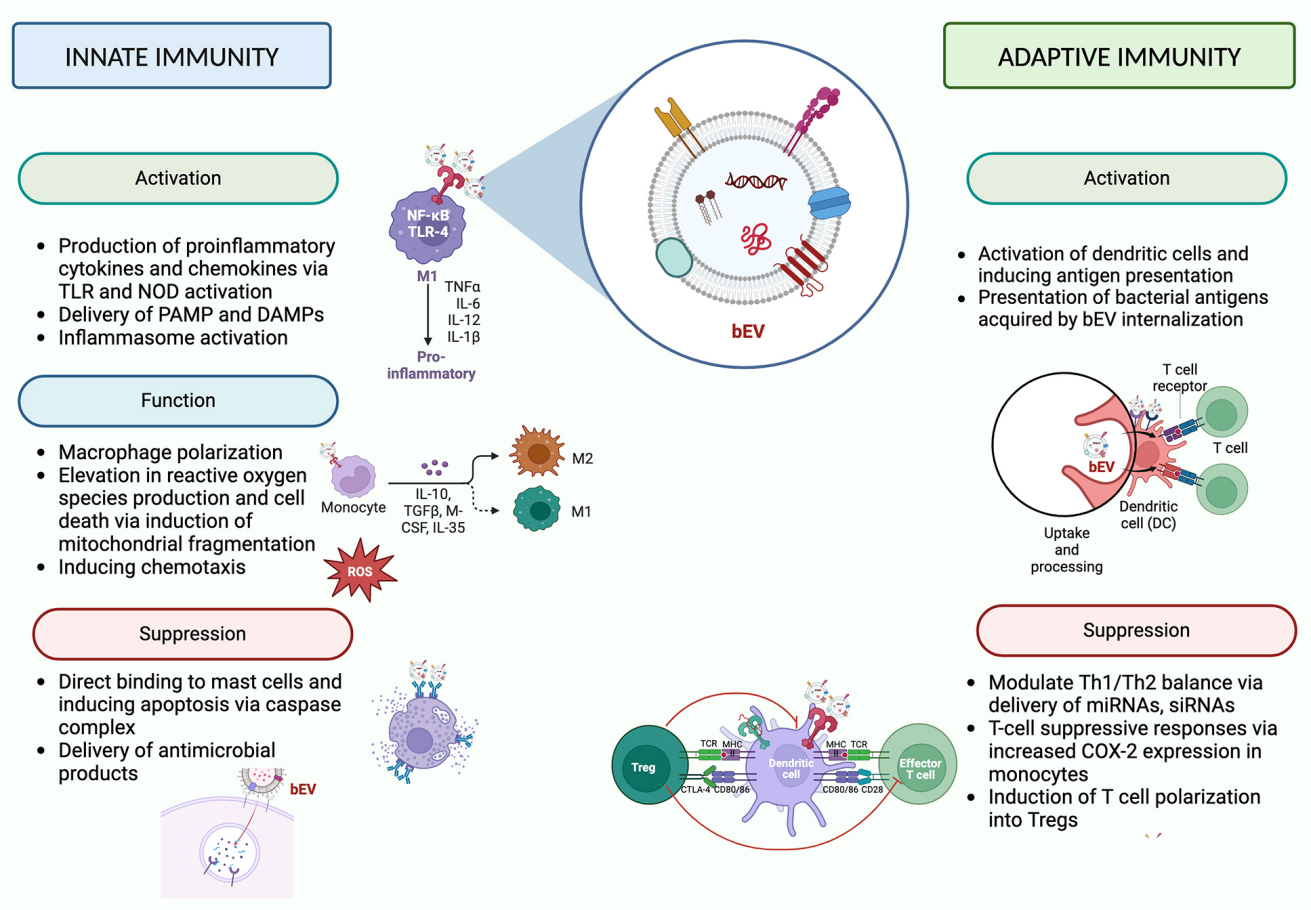
**Effects of bacterial extracellular vesicles on innate and adaptive immunity.** Graphical depictions represent examples of effects attributed to bEVs. DAMPs (damage-associated molecular patterns). Created with BioRender.com.

While there are some broadly-described mechanisms of bEV-host interactions (eg, NFκB activation via LPS/TLR4 [61] and LTA/TLR2 interactions [62]), the complete spectrum of their molecular interactions and associated downstream effects depend on the producing bacteria strain, the recipient cell identity, and the biological context of the interaction (summarized in Figure 1). For example, it has been reported that *E. coli* bEVs elicit the production of TLR4-mediated CXCL8 [76], while *Lactobacillus casei* [78] and *Lactobacillus fermentum* [78] LTA in bEVs stimulates the production of proinflammatory cytokines and chemokines in a TLR2-dependent manner. *Helicobacter pylori* bEVs activate cellular peptidoglycan sensor NOD1, whereas *Pseudomonas aeruginosa* bEVs generate atypical inflammation in human monocytes and mouse macrophages by inflammasome activation, IL-1 production, and cell death via caspase-5 [63, 64]. In addition, bEVs have also been shown to play a role in macrophage polarization. Chen et al reported that *Fusobacterium nucleatum* bEVs aggravated periodontitis by macrophage polarization towards a pro-inflammatory phenotype [65].

In contrast, *Mycobaterium tuberculosis* bEVs suppress the immune response by activation of immunosuppressive macrophages [66]. T-cell suppressive responses are also modulated by *H. pylori* bEVs via increased COX-2 expression in monocytes [63]. *Lactobacillus* bEVs can attenuate LPS-stimulated inflammatory signals in macrophages and microglial cells downstream of Erk and p38 signaling pathways. A similar observation was noted using murine macrophages by *Spirometra erinaceieuropaei* plerocercoid bEVs [67]. These and other studies demonstrate the large breadth of downstream effects that bEVs can mediate on the immune system.

An increasing number of studies are interrogating the role of bEVs at barrier sites, such as lungs, intestine, and sinuses, in the context of infection. For example, bEVs in bronchoalveolar lavage fluid activate cytokines and inflammatory mediators in alveolar macrophages [68], while various strains of *Enterovirus* bEVs are associated with acute gastroenteritis [69]. The bEVs in nasal lavage fluid activate mucin-type O-glycan biosynthesis and the TGFβ pathway in chronic rhinosinusitis [70]. *Acinetobacter baumannii* bEVs are taken up by mammalian cells and activate the host GTPase dynamin-related protein 1 that enhances its accumulation on mitochondria, causes mitochondrial fragmentation, elevation in reactive oxygen species production, and cell death [71].

In addition, bEVs have been shown to be involved in non-infectious diseases, such as autoimmuninty, asthma, and atopic dermatitis. For instance, *Akkermansia muciniphila* bEVs demonstrated protective effects on the dextran sulfate sodium (DSS)-induced model of inflammatory bowel disease (IBD) [72], suggesting bEVs as a potential mediator of commensal microbe-host interaction in this disease. In another study of this IBD model, orally administered bEVs from *Bacteroides thetaiotaomicron* also ameliorated inflammation [73]. In addition to gastrointestinal microbiota, alterations in lung microbiota have been shown to affect mucosal immunity. For example, *Pseudomonas aeruginosa* has been shown to secrete regulatory sRNA52320, a fragment of a *P. aeruginosa* methionine tRNA that has been loaded into bEVs, reducing LPS-induced IL-8 secretion in cultured primary human airway epithelial cells [74]. Using metagenomic analysis, increased levels of the bEVs of *Klebsiella spp* and decreased levels of bEVs from *Lactobacillus*, *Sphingomona*s, *Akkermansia*, and *Micrococcus spp* were found in the serum of patients with asthma [75, 76]. *Lactococcus lactis* bEVs modulate T helper (Th)1/Th2 balance by stimulating dendritic cells in a mouse model of *S. aureus*-induced atopic dermatitis [77]. *Lactobacillus plantarum* EVs demonstrated protection against *S. aureus*-induced atopic dermatitis [78]. *Bifidobacterium* bEVs have been shown to be associated with asthma susceptibility and atopic dermatitis by binding to mast cells and suppressing the allergic reaction inducing mast cell apoptosis [79].

In summary, bEVs from diverse microbial sources have been shown to have potent effects on immunity in a variety of contexts. These intrinsic properties have led to significant interest in the development of bEVs as a therapeutic immunomodulation platform, including for cancer treatment, as discussed below.

### Bacterial EV Applications for Cancer Immunotherapy

While cancer immunotherapy has transformed the treatment of individuals with cancer, not all patients benefit from these approaches [80]. Dysfunctional immune activation and/or redundant mechanisms of anti-tumor immunity exhaustion in the immunosuppressive tumor microenvironment (TME) play fundamental roles in facilitating cancer immune evasion [81]. Thus, strategies that boost immune activity in the TME can be useful to overcome resistance to immunotherapy [82].

Initial efforts to harness microbes for cancer treatment included developing oncolytic bacteria and bacteria expressing cytotoxic proteins or tumor-specific antigens [83, 84]. Accumulating research has demonstrated that several bacterial species can potentially eliminate tumors by direct bacterial cytotoxicity, enhanced host immune response, and production of enzymes, bacteriocins, and toxins that disrupt the proliferation of cancer cells [85]. Attenuated *Salmonella typhimurium* and *Clostridium novy*, which were modified to express HlyE, Stx2, and recA, resulted in stimulation of the host immune response, leading to the activation of cytokines such as IL-2, IL-4, IL-18, and CCL21, ultimately resulting in tumor regression and necrosis in preclinical models of various solid tumors [86, 87]. However, phase 1 clinical trials have failed to show tumor shrinkage in patients with metastatic melanoma, squamous cell carcinoma, and other solid tumors [86, 87]. Mice treated with *Escherichia coli* or *Salmonella typhimurium* strains producing the ClyA toxin were shown in a small number of trials to have reduced tumor development [88, 89]. Nevertheless, several critical obstacles remain in clinical applications of bacteriotherapy in cancer treatment, including bacterial persistence, toxicity, DNA instability, and targeting efficiency [84].

As described in the previous section, bEVs possess immunomodulatory effects that are dependent on the microbial source and target immune cell, which constitute them as cell-free alternatives to bacteriotherapy. Given that essentially all bacteria produce them, bEVs exhibit wide heterogeneity in terms of dimensions, surface elements, and molecular cargo, making this a versatile platform technology for therapeutic development [90]. In addition, bEVs have the capability to traverse biological barriers while maintaining stability in the bloodstream [91, 92]. They also have the potential to deliver their payload with some selectivity to the TME, either by passive accumulation as biological nanoparticles [40, 93], by engineered expression of tumor-targeting ligands [93], or by incorporation into host cells that home to tumors [94]. Furthermore, there is some successful clinical experience with bEVs, exemplified by the MenBVac vaccine for *Neisseria meningitidis* [95]. As a result of these characteristics, harnessing the immunostimulatory properties of bEVs along with additional engineering (such as incorporation of chemotherapeutic drugs and tumor-targeting ligands) comprises an area of intense research efforts [96–98].

### Harnessing Anti-Tumor Effects of Bacterial EVs

While efforts to develop therapeutic bEVs for cancer have utilized a diverse pool of source bacterial strains (summarized in Table 1), many have focused on *E. coli* strains given the availability of relevant reagents and ease of modification. In an early study of bEVs as a potential immunotherapeutic intervention for cancer treatment, Kim et al [40] utilized bEVs derived from *E. coli* that had been genetically engineered to lack the *msbB* gene, resulting in a strain with reduced endotoxin activity.

**Table 1.** Engineered bEVs for Therapeutic Applications in Cancer

bEV source	Engineering method	Treatment model	Treatment effect	Reference
**Loading**				
*E. coli*	Synthetic bEVs generated with lysozyme and high pH treatment, resulting in bEVs with fewer cytosolic components	Melanoma (B16F10) or colon carinoma (CT26 cells)-bearing mice	Tumor growth inhibition, enhanced efficiency of anti-PD1 therapy	[100]
*E. coli* BL21	Functionalizing bEVs with tumor-targeting DNA aptamers	4T1 breast cancer-bearing mice	Selective pyroptosis, increased effector T-cell infiltration, decreased regulatory T cells, suppression of tumor growth	[101]
*E. coli* Nissle 1917	Perhexiline-loaded *E. coli* bEVs	CT26 colon cancer cell line *in vitro*	Repolarization of macrophages to M1 phenotype, apoptosis and inhibition of cell growth, invasion and migration	[102]
*Klebsiella pneumonia*	Doxorubicin-loaded bEVs	A549 lung tumor-bearing BALB/c nude mice	Tumor growth inhibition, recruitment of macrophages into tumor microenvironment	[103]
*E. coli* BL21*(ΔmsbB)*	bEVs loaded with paclitaxel and Redd1 siRNA	Triple-negative breast cancer *in vivo* model (4T1 tumor-bearing mice)	Enhancement of glycolysis in M2 macrophages with polarization, shift in tumor metabolism, suppression of tumor growth, inhibit tumor metastasis	[104]
**Targeting ligands**				
*E. coli*	bEVs expressing an EGFR-targeting scFv version of panitumumab	Triple-negative breast cancer murine models (4T1 tumor bearing mice)	Enhanced tumor targeting, increased M1 macrophages and CD8 T lymphocytes	[105]
*E. coli* strain W3110	bEVs expressing ectodomain of programmed death 1 (PD1)	C57BL/6 mice bearing B16 melanoma cells, Balb/c mice bearing CT26 colorectal cancer cells	Recruitment of cytotoxic T cells and dereased immune inhibitory PD-1/PD-L1 effects, enhanced anti-tumor response	[106]
**Combination of loading and targeting**				
*E. coli* K-12 W3110 strain	bEV-associated HER2-specific antibody in the surface by fusion to Cytolysin A, and loaded with siRNA targeting kinesin spindle protein	Cell lines that overexpress HER2 (SKOV3, BT474, and HCC-1954), HCC-1954 xenografts	Suppressed tumor proliferation *in vitro* and *in vivo*	[107]
Attenuated *Salmonella typhimurium*	bEV coating with DSPE-PEG-RGD and loaded with 5-fluorouracil (5-FU) prodrug tegafur	B16F10 melanoma, 4T1 breast cancer models *in vivo*	Inhibition of tumor growth, decreased metastatic nodules to lungs in melanoma model.	[109]
*Magnetospirillum gryphiswaldense*	bEVs containing iron ions, doxorubicin and modified with DSPE-PEG-cRGD peptides	4T1 breast cancer and drug-resistant MCF-7/ADR tumors in mice	Inhibition of tumor gowth and metastasis to the lung in a 4T1-Luc model of breast cancer metastasis to the lung *in vivo,* ferroptosis of targeted cancer cells	[109]
*E. coli*	miRNA nano-delivery system using zeolitic imidazolate framework-8 (ZIF-8) coated with bEVs that are engineered to display PD1	Murine breast cancer model	Increased tumor treatment efficiency in murine breast cancer models *in vivo* by enhancing immune activation and checkpoint inhibition mediated by bEV-PD1	[110]
*Salmonella typhimurium-*pG^FlaB^	Doxorubicin-loaded *Salmonella* bEVs	Mice bearing C6 glioma	Inhibition of tumor growth, macrophage repolarization, neutrophil recruitment, P-gp downregulation	[94]

The authors demonstrated bEV accumulation in subcutaneous mouse tumor models and the capacity to elicit a durable anti-cancer immune response that leads to substantial reductions in tumor sizes, dependent on CD8+ cells and the presence of IFN-γ [40]. In another study, *E. coli* BL21 (DE3) bEVs effectively induced immune responses, mediated by IL-6, TNF- α, IFN- γ, and CXCL10, which eliminated subcutaneous tumors in mouse models of urothelial and breast carcinoma [99]. Additionally, that study revealed that the administration of bEVs resulted in an increase in the population of intratumoral CD8+ T cells with stem-like properties that specifically target cancer antigens. This augmentation rendered the cancer cells more receptive to the effects of anti-PD-1 antibody immunotherapy. In addition, Won et al have shown that the application of *E. coli* bEVs in the TME resulted in the infiltration of T lymphocytes that specifically target cancer cells and express exhaustion markers, such as PD-1, TIM3, and CD39 [99], suggesting the potential for combination with checkpoint inhibitors that are in clinical use or under development.

In addition, the investigation of the application of bEVs from other species is ongoing. *Akkermansia muciniphila* bEVs were shown to have anti-tumor effects in a syngeneic prostate cancer mouse model by increasing the CD8+ lymphocytes and promoting macrophage differentiation into an inflammatory phenotype [111]. Bacterial EVs from other gram-negative strains (*Salmonella enterica*) and gram-positive sources (*Lactobacillus acidophilus* and *Staphylococcus aureus*) also showed anti-cancer effects in mice with CT26 tumors [40]. In addition, bEVs derived from *Bacillus licheniformisin* inhibited the tumor growth both in MDA-MB-231 breast cancer and A549 lung cancer cell lines [112]. These studies suggest that diverse speceies' bEVs could have beneficial immunomodulatory properties for cancer therapy.

Despite these promising results, there is still the concern that bEV engagement of TLRs will lead to severe systemic toxicity. Some groups have approached this challenge by modifying the parental bacteria to attenuate endotoxin levels in bEVs [40, 107]. Others performed additional processing of purified bEVs with high pH treatment resulting in lower quantities of bacterial protein, RNA, and DNA [100]. While these bEVs exhibited an inability to induce an immunologic response through TLR3, 7, 8, and 9, they retained their ability to serve as adjuvants for a mouse melanoma-derived EV-based vaccine, suppressing tumor growth and metastasis and enhancing the treatment effect of anti-PD-1 antibodies [100]. Yet another approach has been to shield endotoxin with tumor-targeting DNA aptamers. In one application, this enabled triggering LPS-dependent pyroptosis in preclinical breast cancer models without severe systemic toxicity [101].

Direct experimental evidence in cancer models or extrapolation from microbiology and infectious disease research suggests that the anti-tumor effects of bEVs are largely driven by interaction between bEV components with innate immune receptors. However, there is also evidence that bEVs can directly impact cancer cell biology. For instance, *Akkermansia muciniphila* bEVs can transfer bacterial acetyltransferase to both colorectal cancer cell lines and mouse cancer models, resulting in increased H3K14 acetylation and heat-shock protein 70 (Hsp70) expression, which indirectly induces immune activation of CD8+ cytotoxic T lymphocytes [113]. The bEVs produced by *E. coli* strain A5922 were shown to induce oxidative stress, increase PINK1 expression, and reduce mitochondrial membrane potential in HT-29 colon cancer cells, which led to decreased viability [92]. In HepG2 hepatic cancer cells, *Lactobacillus rhamnosus* GG bEVs also resulted in a higher Bax/Bcl-2 ratio with activation of apoptosis and subsequent cell death *in vitro* [114]. Direct effects on cancer cells may also synergize with cancer-directed therapies, as was the case with *Klebsiella pneumoniae* bEVs that enhanced the anti-cancer properties of tamoxifen in MCF-7 cells via the activation of Cyclin E2 and pERK signaling, which resulted in inhibition of cancer cell proliferation *in vitro* [115].

Despite being considered potential innovative therapeutic tools, there are several questions regarding the optimal dose, delivery method, biodistribution, and pharmacokinetics of bEVs. Until now, the majority of pre-clinical investigations have focused on examining the biodistribution and pharmacokinetics of EVs through the utilization of mouse models. Various biodistribution studies demonstrated that bEVs are primarily found in organs with a dense vascular network and other tissues connected to the reticuloendothelial system, such as liver, spleen, lung, and kidneys [116]. Furthermore, in the case of mammalian EVs, half-life in blood varies by cell source, ranging from 30 minutes to 6 hours before being cleared by reticuloendothelial macrophages, which detect negatively charged phosphatidylserine on the membrane of EVs [117–119]. Thus, it is possible that the source species will also impact the pharmacokinetics of bEVs. The impact of the administration method on the circulation time and biodistribution of bEVs remains poorly comprehended, as only a limited number of research projects have explicitly evaluated different administration routes, mostly utilizing mammalian cell-derived EVs administered in mice [120]. Although the animal studies offer useful insights into the biodistribution of EVs, these findings may not be completely applicable to bEVs. Variations in extracellular protein/lipid composition, EV labeling technique, and the type of EV donor cells may all have a role in EV biodistribution beyond the initial route of delivery. Circulation times may also be influenced by species distinctions between the recipient and the EV source [121].

Given the rapid elimination of bEVs from circulation, substantial research is needed to design optimal bEV therapeutics able to reach target tissues. One such approach involves the direct delivery of bEVs to tumors for local and/or abscopal therapeutic effects [122]. Additional strategies may include transient blockade of rapid clearance by the reticuloendothelial system, previously shown to enhance tumor accumulation of administered mammalian EVs [119]. Finally, applications of bEVs from some species may have more favorable pharmacokinetic profiles than others. As described in the section below, a different approach could involve engineering to alter the intrinsic properties of bEVs. This area of research is far more advanced in the field of mammalian EVs and synthetic nanoparticles [123–126], which could provide significant insight for bEV applications as well. For example, mammalian EV circulating time was significantly increased when they were engineered to express the “don't-eat-me” signal, CD47, which inhibits phagocytosis by macrophages upon ligation with its receptor, SIRPα [127]. Others have modified the surface of EVs and nanoparticles by chemical modification (click chemistry), glycan modification, and insertion of specific peptides to further modify their pharmacokinetic characteristics [128]. In summary, combinations of optimized administration and engineering strategies are likely to be needed for the effective application of bEVs in cancer therapy.

#### Engineering bEVs for therapeutic applications in cancer.

Aside from having inherent biological activity, bEVs can be further modified to construct customized drug delivery systems either by engineering the producing bacteria or isolated bEVs.

Various methodologies have been devised to encapsulate exogenous proteins and pharmaceutical agents into bEVs, often applying technologies developed for synthetic nanoparticles. These approaches have demonstrated the ability of bEVs to acquire supplementary biological attributes [129]. *Escherichia coli* Nissle 1917 bEVs passively loaded with perhexiline delivered this compound to macrophages, which were reprogrammed from an immunosuppressive to an inflammatory phenotype [102]. When these reprogrammed macrophages were co-cultured with CT26 colon cancer cells, they promoted apoptosis and inhibited invasion and migration [102]; bEVs generated from attenuated *Klebsiella pneumonia* passively loaded with doxorubicin also induced cell apoptosis, cytotoxicity, and immunogenicity by attracting macrophages in the TME of mice with a transplantable model of non-small-cell lung cancer [103]. In a mouse model of triple-negative breast cancer, bEVs from *E. coli* BL21 were surface-functionalized with paclitaxel and electroporated with Redd1 siRNA and were effective immune activators and drug carriers [104]. The absorption of paclitaxel by immunosuppressive macrophages was shown to enhance glycolysis [104]*,* and the use of siRNA to repolarize tumor-associated macrophages (TAM) and increase tumor immune activation led to a shift in tumor metabolism and the subsequent suppression of tumor growth. In another application, “self-blockade” of tumor cell PD-L1 and improved treatment effectiveness in mice tumor models were achieved by the introduction of an encapsulated plasmid expressing PD-1 to cancer cells [130].

Besides loading therapeutics, several groups have developed engineered bEVs with targeting ligands to enhance specificity of delivery. Adriani et al targeted triple-negative breast cancer murine models using modified *E. coli* bEVs expressing an EGFR-targeting scFv version of panitumumab [105]. These scFv-bEVs bound to EGFR-overexpressed cancer cells more than control bEVs and also displayed enhanced tumor targeting in EGFR-expressing models *in vivo* [105]. Similar to prior studies, these bEVs re-polarizated macrophages to an inflammatory phenotype and potentiated the recruitment of cytotoxic CD8+ T lymphocytes [105]. Engineered *E. coli* strain W3110 bEVs with surface PD-1 ectodomain also demonstrated potent anti-tumor responses *in vivo* [106]. The modified PD-1-bEVs attach to PD-L1 on the surface of cancer cells, causing it to be internalized and decreasing surface expression, therefore shielding attracted T cells from immune suppressive signals of the PD-1/PD-L1 axis [106].

As technologies for loading and targeting are expanding, studies are beginning to combine the 2 approaches for further enhancement of therapeutic effects. For example, the *E. coli* K-12 W3110 strain was modified to present bEV-associated HER2-specific antibody on the surface by fusion to Cytolysin A and loaded with siRNA targeting kinesin spindle protein [107]. These modified bEVs exhibited cytotoxic effects on cell lines that overexpress HER2 (SKOV3, BT474, and HCC-1954) when tested *in vitro*. Additionally, *in vivo* experiments using HCC-1954 xenografts have shown that bEVs significantly suppressed tumor development compared to the control group treated with a vehicle. Others have tested the use of engineered bEVs in combination with synthetic nanomaterials to generate hybrid nanoformulations. Chen et al modified attenuated *Salmonella* bEVs to display RGD peptide by functionalization with DSPE-PEG-RGD for cellular targeting [108]. They then combined these bEVs with polymeric micelles encapsulating the 5-fluorouracil (5-FU) prodrug tegafur by extrusion. This hybrid nanoformulation further enhanced tumor cytotoxicity and suppressed tumor development and metastatic nodules in several preclinical mouse tumor models [108]. DSPE-PEG-RGD-based targeting was also employed on bEVs from the magnetotactic bacterium *Magnetospirillum gryphiswaldense*, cultured in the presence of iron and doxorubicin to generate bEVs loaded with all 3 of these components [109]. These bEVs resulted in ferroptosis of targeted cancer cells and immune activation and displayed preclinical therapeutic efficacy in primary and metastic breast cancer models [109]. Cui et al also generated a hybrid nanoformulation consisting of miRNA encapsulated in a zeolitic imidazolate framework-8 and then coated with *E. coli* bEVs expressing surface PD-1/Cytolysin A fusion protein as a tumor-targeting ligand [110]. These hybrid nanoparticles effectively delivered siRNA intravenously to mouse breast cancer models *in vivo*, significantly delaying tumor growth [110]. In another study, Mi et al employed a tandem bacteriotherapy/bEV strategy to target brain tumors, a long-standing challenge in drug development, given the blood-brain barrier [94]. Specifically, they showed that bacteriotherapy of brain tumors with attenuated Salmonella enhanced accumulation of *Salmonella* bEVs loaded with doxorubicin via a hitchhiking process on circulating neutrophils (ie, bEVs associated with neutrophils in circulation, which the authors claim helped bring them to tumors) [94]. Given the complexity of this strategy, it remains to be seen whether such approaches will find broader application.

#### Bacterial EVs in cancer vaccine research.

Several bEV-based acellular vaccines have previously established the potential of bEVs to generate an adaptive memory immune response [131]. The most notable examples are vaccines against *Neisseria meningitidis* serogroup B, of which there are clinically used products [132, 133]. In addition, several studies have demonstrated the efficacy of bEVs derived from several gram-positive bacteria, such as *Clostridium perfringens*, *Streptococcus pneumoniae*, *Bacillus anthracis, Mycobacterium tuberculosis, and Staphylococcus aureus,* as vaccines against infections in mouse models [134]. These bEVs have been shown to significantly improve survival rates following exposure to a lethal challenge with corresponding bacteria in preclinical models [134].

In the last decade, bEVs have been proposed as an appealing candidate for cancer vaccines owing to their notable immunogenicity, lack of proliferative capacity, and ability to transport and display custom target antigens on their membranes [135, 136]. These bEV cancer vaccines typically involve genetic engineering to introduce compounds such as foreign proteins and small RNAs into the vesicle lumen or onto the membrane surface, where they can act as antigens to stimulate an immune response while preserving the vaccine's natural immunogenicity and minimizing any potential adverse effects [135]. For instance, Grandi et al demonstrated that administration of *E. coli* bEVs, engineered to express an epitope of the tumor-enriched antigen FAT1, generated tumor-targeting antibodies that protected mice from CT26 murine colon adenocarcinoma [137]. The same group also developed *E. coli* bEVs expressing peptide epitopes of EGFR-EGFRvIII by fusion to the *Neiserria meningitidis* factor H binding-vIII protein (Nm-fHbp) and described a vaccine-induced antibody response that was associated with inhibition of EGFRvIII-expressing B16F10 tumor growth in mouse models [138]. Moreover, bEVs have also been used to develop auto-antibodies against tumor promoting growth factors, such as FGF, which also provided protection against tumor growth in preclinical models [139].

To facilitate facile coating of bEVs with diverse tumor antigens, Cheng et al developed bEVs expressing Cytolysin A fused to SpyCatcher and SnoopCatcher peptides, which form covalent bonds with antigens fused to SpyTag and SnoopTag peptides, respectively [140]. In their proof-of-concept investigations, the researchers demonstrated this methodology can induce T-cell reactions against several tumor antigens, resulting in a significant impairment in tumor growth in mouse models of lung cancer [140]. Similarly, Li et al developed engineered bEVs expressing the RNA binding protein L7Ae, which allowed for post-purification binding to select mRNA vaccine constructs for tumor antigens [141]. Plug-and-play approaches such as these could comprise useful platforms for the rapid generation of personalized vaccines using off-the-shelf bEVs and custom-synthesized peptide or mRNA pools.

In addition to custom immunogen loading, others have further modified bEVs to enhance their efficacy as vaccines or combination vaccine/therapeutics. They incorporated the lysosomal escape protein listeriolysin O to facilitate and enhance the expression of the delivered mRNAs after cellular uptake [141]. In another combination approach, Liang et al developed a trained-immunity-related vaccine using bEVs expressing SIRPα to facilitate phagocytosis by antigen-presenting cells and GM-CSF to further activate these cells [142]. They showed that this vaccine, produced using *E. coli* bEVs, reduced subcutaneous tumor development in 2 models: MC38 (TAM-rich with low in T cells) and B16-F10 (T cell-rich with low TAM). They also reported that MC38 antitumor mechanisms relied on both trained innate immunity and activated T-cell response, whereas B16-F10 responses predominantly relied on trained innate immunity, revealing that modulating macrophage activity within tumors is essential for vaccine-induced immunotherapy [142].

As in the case of immunotherapeutic bEVs, further understanding of bEV-immune interaction mechanisms will be instrumental in the further development of this platform as a form of novel cancer vaccines.

## DISCUSSION: PATHWAY TO THE CLINIC

**Figure 2.**
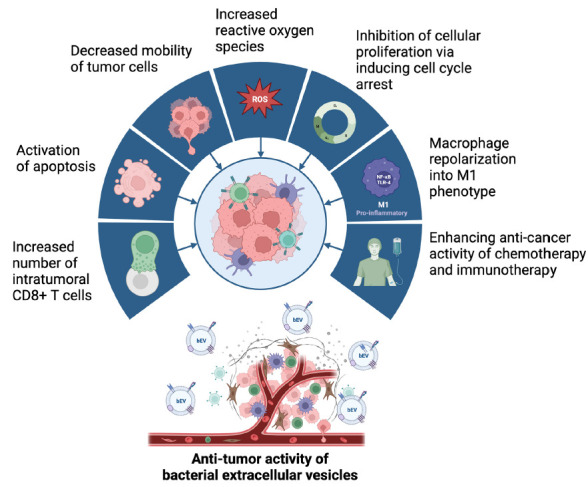
**Proposed mechanisms of action of bacterial extracellular vesicles with anti-tumor effects observed in preclinical models.** Created with BioRender.com.

The understanding of the spectrum of bEV biological effects is facilitating the development of innovative therapeutic technologies; bEVs possess the capability to transfer biological cargo such as DNA, RNA, proteins, and lipids, providing at least partial protection against breakdown by the host organism [143]. The ability to tailor the interactions and functionalities of these biological nanoparticles suggests that bEVs may find use in cutting-edge diagnostic and therapeutic applications [96]. The anti-tumor effects demonstrated with the use of bEVs in preclinical cancer models (Figure 2) capitalize on a range of features, including inherent immunomodulatory capabilities, favorable potential for large-scale production, adaptability for personalized therapies, and enhanced safety profiles [144].

Despite these early promising results, there exist several significant hurdles in the transition of bEVs from bench-to-bedside clinical applications, some of which were summarized in position papers of the International Society for Extracellular Vesicles [145] and Chinese Society for Extracellular Vesicles [146]. These concerns include toxicity of bacterial components, consistency of purification, and cost of production.

Regulatory agencies generally classify bEV-based treatments as biologics [147]. Although bEVs exhibit reduced susceptibility to post-purification alterations in comparison to their progenitor cells, their analytical characterization and manufacturing processes pose more complexities when compared to other biologics often employed in clinical settings, such as monoclonal antibodies, in particular, due to their heterogeneity in size, origin, and composition. Indeed, regulatory clearance will likely require that bEVs meet certain purity standards during manufacturing in addition to showing efficacy and safety [148]. Given the breadth of possible bEV sources and engineering modifications, it is likely that some of these will have to be determined on a case-by-case basis. Still, one could envision certain common criteria (for example, endotoxin activity or lack of high-molecular-weight, non-vesicular component contamination, such as gram-negative bacterial fimbriae [149] or the Factor H binding protein from *Neisseria*) [150]. Some of these criteria may need to comprise part of even early preclinical development to avoid investment of resources in candidate therapeutic bEVs that do not have beneficial properties.

Despite any process standardization efforts, challenges with the concept of bEV purity will most likely remain. No current technologies exist that can produce “pure” EVs [151], while technologies that come closest (such as immunoisolation) [152] are not practically scalable. Moreover, discoveries such as that of a protein corona adsorbed to the surface of EVs with functional ramifications [153] raises the possibility that more purity may not always be beneficial. The biophysical overlap of putative contaminants with subsets of the heterogeneous EVs poses additional challenges, as methods that deplete one may affect the relative makeup of the other. In summary, perhaps more so than in the case of recombinant proteins, the industrial phrase “the process is the product” is highly applicable to the development of bEV therapeutics. In practice, dealing with this challenge may involve testing the impact of different bEV-enrichment strategies on desired functional attributes and toxicity of candidate products [145]. This approach goes together with a need for method standardization and transparency (for example, through EV-TRACK platform reporting) [154]. In addition, once methods are standardized for a given product, multiple orthogonal metrics of purity can be employed. These could include enrichment of total vesicular particles (measured by methods such as nanoparticle tracking analysis, tunable resistive pulse sensing, magnetic resistive pulse sensing, or nano-flow cytometry) [155]; particle-to-protein ratio [156]; enrichment of target marker proteins; and/or depletion of established undesired contaminants for a given bEV source. Finally, given the invariable presence of non-vesicular components in preparations, it is important to link function specifically to the bEVs themselves as opposed to the contaminants. Experimental approaches to this are still evolving and could include things like loss-of-function upon treatment with detergents, retaining function upon treatment with proteases/nucleases, or retaining function in small-scale preparations of the highest purity possible.

Regarding mass production of bEVs, a significant benefit of a bacterial source as opposed to developing therapeutic EVs from a mammalian cell source is the ease of scaling up the process (usually in the form of bioreactor cultures). Several studies have described ways of improving bEV yields in these settings by additional environmental stresses including high pressure, high temperature, nutritional deficiency, shear stress, and ethanol [157, 158]. It remains to be seen whether these strategies will retain the desired functionality of purified bEVs. Regarding downstream purification, most development involves employing tangential flow filtration, followed by size exclusion or affinity chromatography [149, 159]. Recently, Won et al piloted a modified large-scale manufacturing process by combining metal precipitation and size flexclusion chromatography, which succeeded in cost-effectively mass-producing *E. coli* bEVs [99]. They compared the results of this novel technique to those obtained by using more traditional approaches with ultracentrifugation and buoyant density gradient ultracentrifugation [40, 160], observing yield and purity at least as good as those observed with other less scalable technologies. These studies comprise important first steps in developing workflows for efficient mass production of bEVs for further clinical development.

While past experience with bacteria-derived therapeutics and ongoing research are likely to overcome these technical challenges, there is still much to learn about the pharmacologic properties of bEVs. At a fundamental level, it is difficult to *a priori* predict the biological effects of bEVs, some of which can even have immunosuppressive properties [161]. In several published studies, the results of *in vivo* preclinical experiments are in line with the biological effects of bEVs modeled through *in vitro* functional assays [162, 163]. Applications of technologies used in the synthetic nanoparticle and mammalian EV fields can further improve the ability to predict and modulate the properties of bEVs. For instance, by modifying nanoparticles to incorporate fluorescent markers, researchers can see the gradual incorporation and movement of bEVs into immune cells or organ-on-chip models as they happen in real-time [164]. Magnetic nanoparticles can also change bEV's spatial distribution, allowing researchers to study how their localization influences human-cell interaction [165]. Moreover, encapsulating bEVs in synthetic nanoparticles may improve their stability and bioavailability [166]. Combining these strategies may comprise promising solutions to optimize the pharmacologic properties of bEVs.

A vast majority of *in vivo* studies have utilized mouse models, and it remains unclear how other mammalian (especially primate) immune systems will respond to these complex agents. Given the established distinct characteristics of murine immune systems [167, 168], preclinical testing in non-human primates is likely to represent a critical step in the development of therapeutic bEVs for at least some applications. This is true both to elucidate on-target immune effects and to assess toxicity, especially as multiple groups are working on engineering “attenuated” bacteria that produce bEVs with presumed less systemic toxicity [108, 169, 170].

Another major concern is the development of anti-drug antibodies or other forms of anti-drug immune response against a repeatedly administered xenobiotic drug. Long-term, repetitive administration of non-human EVs can result in either immunogenicity or allergenicity [171, 172]. Qing et al attempted to prevent the formation of anti-drug antibodies by encapsulating *E. coli* bEVs in biocompatible calcium phosphate which dissolves in acidic TME to release the bEV. When paired with a photosensitizer, these encapsulated bEVs elicited photothermal immunogenic cell death in CT26 solid tumor mice and 4T1 tumor mice [173]. It remains to be seen whether engineering approaches such as these are necessary to retain therapeutic properties of bEVs administered repeatedly.

## CONCLUDING REMARKS

Studies investigating potential therapeutic applications of bEVs for cancer immunotherapy have been significantly increasing over the years, with promising results. However, if they are to be further developed as a therapeutic platform, our understanding of their biologic and pharmacologic properties in model systems and primates will need to significantly expand. The same is true for identifying bEV sources with desirable intrinsic properties and/or designing engineering modifications to improve the safety and efficacy of potential therapeutic bEVs. Elucidating mechanisms of biogenesis, heterogeneity, and downstream molecular signaling on tumor and microenvironment cells is critical for taking this technology to more advanced stages of drug development. Moreover, this will enable the rational combination of bEV therapeutics with other modalities, such as various immunotherapy approaches. Advancements in understanding these biological parameters, together with improvements and standardization of technologies to manufacture and characterize bEVs and their pharmacologic properties will help transform this field from proof-of-principle to actual clinical application as a versatile and promising therapeutic platform for cancer.
